# Supplementary material for: "The added value of 18f-FDG PET/CT in the assessment of onset and steroid resistant polimyalgia rheumatica"
Source: PLoS One. 2021 Sep 24;16(9):e0255131. doi: 10.1371/journal.pone.0255131 (PMC8462716; doi:10.1371/journal.pone.0255131)
Supplement: S1 File — (PDF) [file pone.0255131.s001.pdf]

GENDEI	PMR TYPE	Symptoms Start Date	ASTHENI/	WEIGHTLOSS
F	Start PMR	Sep-10	yes	0
F	Steroid Resistant	Jun-11	No	0
F	Steroid Resistant	Jun-08	yes	0
F	Start PMR	Mar-11	yes	0
F	Start PMR	Sep-11	yes	0
F	Steroid Resistant	Jun-09	No	0
M	Start PMR	Apr-12	yes	yes
F	Start PMR	Apr-12	No	yes
F	Start PMR	May-11	yes	yes
F	Steroid Resistant	Nov-11	yes	0
M	Start PMR	Jun-12	No	0
F	Steroid Resistant	Jun-09	yes	0
F	Start PMR	Feb-12	yes	0
M	Start PMR	Jun-12	yes	0
F	Steroid Resistant	Jun-95	yes	0
F	Steroid Resistant	Dec-14	No	0
F	Start PMR	Feb-11	yes	0
F	Start PMR	Aug-15	yes	yes
M	Steroid Resistant	Dec-10	No	0
F	Start PMR	Apr-12	yes	0
F	Steroid Resistant	Jun-02	No	0
F	Start PMR	Mar-13	yes	0
F	Start PMR	Dec-10	No	0
F	Steroid Resistant	Jun-03	No	0
F	Start PMR	Jun-15	No	0
F	Steroid Resistant	Jun-13	yes	0
F	Steroid Resistant	Jun-15	No	0
M	Start PMR	Jun-14	No	0
F	Steroid Resistant	Jun-09	yes	0
F	Steroid Resistant	Sep-13	No	0
M	Start PMR	Jun-11	yes	0
M	Start PMR	Jul-16	yes	yes
F	Steroid Resistant	Jun-03	No	0
F	Start PMR	Jun-04	yes	0
F	Steroid Resistant	May-11	yes	0
F	Start PMR	Jun-08	yes	0
F	Start PMR	Oct-16	yes	yes
M	Start PMR	Apr-17	yes	yes
M	Steroid Resistant	Apr-11	No	0
M	Start PMR	Jun-13	yes	0
F	Steroid Resistant	May-13	No	0
F	Steroid Resistant	Jun-12	No	0
F	Start PMR	May-15	yes	yes
F	Start PMR	Apr-17	yes	yes
M	Start PMR	Jan-12	yes	0
F	Start PMR	Jul-12	yes	yes
F	Steroid Resistant	Jun-11	yes	0
M	Steroid Resistant	Jul-13	No	0

F	Steroid Resistant	Apr-14	yes	yes	
M	Steroid Resistant	Jan-12	yes		0
F	Start PMR	Nov-14	No		0
M	Start PMR	Jan-12	yes		0
M	Start PMR	Jun-10	yes	yes	
F	Start PMR	Sep-15	yes	yes	
F	Steroid Resistant	Jun-00	yes		0
F	Start PMR	Jun-15	No		0
F	Steroid Resistant	Jun-11	yes	yes	
M	Start PMR	Apr-11	yes	yes	
M	Steroid Resistant	Mar-10	No		0
F	Start PMR	Jun-11	No		0
F	Steroid Resistant	Feb-11	yes	yes	
M	Start PMR	Jun-10	yes	yes	
M	Steroid Resistant	Jun-00	No		0
F	Start PMR	Nov-16	No		0
F	Steroid Resistant	Jun-01	yes	yes	
F	Start PMR	Jul-11	yes	yes	
F	Steroid Resistant	May-16	yes		0
F	Steroid Resistant	Jun-09	yes	yes	
F	Steroid Resistant	Oct-14	No		0
F	Start PMR	Jul-11	yes	yes	
F	Steroid Resistant	Mar-15	yes		0
F	Steroid Resistant	Mar-15	yes	yes	
F	Steroid Resistant	Jun-13	No		0
F	Steroid Resistant	Apr-10	yes	yes	
M	Steroid Resistant	Aug-11	No		0
F	Steroid Resistant	Nov-13	No	yes	
M	Start PMR	Aug-13	yes	yes	
M	Steroid Resistant	Nov-09	yes		0
F	Steroid Resistant	Jun-10	yes	yes	
F	Steroid Resistant	Aug-17	No		0
M	Steroid Resistant	Nov-15	No		0
M	Start PMR	Apr-13	yes	yes	
F	Steroid Resistant	Jun-08	yes		0
F	Start PMR	Nov-10	yes	yes	
F	Start PMR	May-11	yes	yes	
M	Start PMR	Mar-16	yes	yes	
M	Start PMR	Jan-12	No		0
F	Steroid Resistant	Sep-09	No		0
F	Steroid Resistant	Jun-09	No		0
F	Start PMR	Oct-11	No	yes	
F	Steroid Resistant	Feb-17	yes	yes	
F	Steroid Resistant	Nov-18	No		0
F	Start PMR	Oct-10	yes		0
F	Steroid Resistant	Jun-10	No		0
M	Steroid Resistant	Jun-13	yes	yes	
F	Start PMR	Feb-11	yes	yes	
M	Start PMR	Jul-12	yes	yes	
F	Steroid Resistant	Mar-17	yes	yes	

F	Start PMR	Apr-17	yes		0
M	Start PMR	Dec-12	yes		0
F	Start PMR	Sep-13	yes	yes	
F	Start PMR	Apr-17	No		0
M	Steroid Resistant	Jul-17	yes	yes	

FEVER	MORNING RIGIDITY	CERVICAL PAIN	SCAPULAR WAIST PAIN	PELVIC WAIST PAIN
yes	No	yes	yes	yes
No	No	No	yes	No
No	yes	yes	yes	yes
No	No	yes	yes	yes
No	yes	yes	yes	yes
yes	yes	yes	yes	yes
No	yes	No	yes	yes
No	yes	No	yes	yes
No	No	yes	yes	yes
No	No	No	No	No
yes	yes	yes	yes	yes
No	yes	yes	yes	yes
No	No	yes	yes	yes
No	yes	No	yes	yes
No	No	yes	yes	yes
No	No	yes	yes	yes
No	No	yes	yes	yes
No	yes	yes	yes	yes
No	yes	yes	yes	yes
yes	yes	yes	yes	yes
No	yes	No	yes	yes
No	yes	No	yes	yes
No	No	yes	yes	yes
No	No	No	No	No
No	No	yes	yes	yes
No	yes	yes	yes	yes
yes	yes	yes	yes	yes
No	No	No	yes	yes
No	No	yes	yes	yes
No	yes	yes	yes	No
No	yes	yes	yes	yes
No	No	yes	yes	yes
No	No	yes	yes	yes
No	No	yes	yes	yes
No	No	yes	yes	yes
No	No	No	yes	yes
yes	yes	yes	yes	yes
yes	No	No	yes	No
No	yes	yes	yes	yes
No	No	yes	yes	yes
No	yes	yes	yes	yes
No	yes	yes	yes	yes
yes	yes	yes	yes	yes
No	No	No	yes	yes
No	yes	No	yes	yes
yes	yes	yes	yes	yes
No	No	yes	yes	yes
No	No	yes	yes	No

No	No	No	yes	yes
No	No	yes	yes	yes
yes	No	No	yes	No
No	No	No	yes	yes
No	yes	yes	yes	yes
No	No	yes	yes	yes
No	No	yes	yes	yes
No	No	yes	yes	yes
No	yes	yes	yes	yes
No	yes	yes	yes	yes
No	yes	yes	yes	yes
No	No	No	yes	yes
No	No	yes	yes	No
No	No	yes	yes	yes
No	No	No	yes	No
No	No	yes	yes	yes
No	No	No	yes	yes
No	yes	yes	yes	yes
No	No	yes	yes	yes
No	No	yes	yes	yes
No	No	No	yes	No
yes	yes	No	yes	yes
No	No	yes	yes	yes
No	No	No	yes	yes
No	yes	yes	yes	yes
No	yes	yes	yes	yes
No	yes	yes	yes	No
No	yes	yes	yes	yes
No	yes	yes	yes	yes
No	yes	yes	yes	yes
No	yes	yes	yes	yes
No	No	No	yes	yes
No	No	yes	yes	yes
yes	yes	yes	yes	yes
yes	yes	yes	yes	yes
No	No	yes	yes	yes
No	yes	yes	yes	yes
No	No	No	No	No
No	yes	No	yes	yes
No	No	yes	yes	yes
No	yes	yes	yes	yes
No	yes	yes	yes	yes
No	No	No	yes	yes
yes	No	yes	yes	yes
No	yes	yes	yes	yes
No	No	No	yes	No
No	No	yes	yes	yes
No	yes	yes	yes	yes
No	yes	yes	yes	yes
No	No	No	yes	yes

No	yes	yes	yes	yes
yes	yes	No	yes	yes
yes	No	No	yes	No
No	No	No	yes	yes
No	yes	No	yes	yes

JOINTS	HEADACH	AMAUROyes	MANDIBULAR CLAUDICATIO	PAIN TEMPORAL ARTERY
No	No	No	No	No
No	No	No	No	No
No	No	No	No	No
No	No	No	No	No
yes	No	No	No	No
No	yes	No	No	No
yes	No	No	No	No
yes	No	No	yes	No
No	yes	No	No	No
No	No	No	No	No
No	No	No	No	No
No	yes	No	No	No
No	yes	No	No	yes
No	No	No	No	No
No	yes	No	No	No
yes	No	No	No	No
yes	yes	yes	No	No
yes	No	No	No	No
No	No	No	No	No
No	No	No	No	No
yes	No	No	No	No
No	No	No	No	No
No	yes	No	No	No
No	No	No	No	No
yes	No	No	No	No
yes	No	No	No	No
yes	yes	No	No	No
yes	No	No	No	No
No	No	No	No	No
No	No	No	No	No
No	No	No	No	No
No	No	No	No	No
No	No	No	No	No
No	No	No	No	No
No	No	No	No	No
No	No	No	No	No
yes	yes	No	No	No
yes	No	No	No	No
yes	No	No	No	No
No	No	No	No	No
No	No	No	yes	No
No	yes	No	yes	No
No	yes	No	yes	No
No	No	No	No	No
yes	No	No	No	No
No	No	No	No	No
yes	No	No	No	No
No	yes	yes	No	No

yes	yes	No	yes	No
No	No	yes	No	No
No	yes	No	No	No
No	No	No	No	No
No	No	No	No	No
No	yes	No	yes	yes
No	No	No	No	No
No	yes	No	No	No
No	No	No	No	No
No	No	No	No	No
yes	yes	yes	No	No
yes	No	No	yes	No
No	No	No	yes	No
No	yes	No	No	No
No	No	No	No	No
No	No	No	No	No
No	yes	No	No	No
No	No	No	No	No
yes	No	No	No	No
No	No	No	No	No
yes	No	No	No	No
No	No	No	No	No
No	No	No	No	No
No	No	No	No	No
No	No	No	No	No
No	yes	No	No	No
No	No	No	No	No
No	No	No	No	No
No	No	No	No	No
No	No	No	No	No
No	No	No	No	No
yes	No	No	No	No
No	No	No	No	No
No	No	No	No	No
No	yes	No	No	No
No	No	No	No	No
No	No	No	No	No
yes	No	yes	No	No
No	No	No	No	No
No	No	No	No	No
yes	No	No	No	No
No	No	No	No	No
No	No	No	No	No
No	No	No	No	No
No	No	No	No	No
No	No	No	No	No
No	yes	No	yes	No
No	No	No	No	No
No	No	No	No	No
No	No	No	No	No

No	yes	No	No	No
No	No	No	No	No
No	No	No	No	No
yes	No	No	No	No
yes	No	No	No	No

BLOOD TEST	CRP	GSV	HB	DATA_PET	PDN_DOSIS_PET
4-Oct	110,00	110	84,0	Oct-10	50,0
21-Oct	2,40	33	123,0	Oct-14	2,5
30-Nov	12,00	85	121,0	Dec-11	0,0
1-Jul	1,50	65	107,0	Aug-11	15,0
3-Oct	32,00	51	116,0	Oct-11	0,0
21-Dec	13,00	80	126,0	Dec-11	2,5
23-May	1,90	34	128,0	Jun-12	0,0
30-Jan	159,00	82	99,0	Feb-13	30,0
28-Mar	132,00	105	110,0	Mar-12	0,0
24-May	8,40	50	125,0	May-12	10,0
19-Apr	108,00	49	149,0	Apr-12	0,0
13-Dec	31,00	80	109,0	Dec-11	7,5
8-Mar	0,70	28	126,0	Mar-12	40,0
31-May	4,30	80	129,0	Jun-13	5,0
8-Nov	11,80	67	127,0	Jan-13	5,0
26-Mar	5,90	28	108,0	Apr-15	20,0
24-Apr	28,00	75	134,0	May-11	0,0
12-Nov	5,30	49	131,0	Apr-16	0,0
18-Jun	0,90	2	159,0	Jul-15	5,0
27-Jun	6,00	31	124,0	Jul-12	20,0
26-Mar	114,00	120	101,0	Mar-18	5,0
23-May	24,90	50	118,0	May-13	10,0
4-Oct	94,00	55	101,0	Oct-11	25,0
20-Aug	6,40	36	122,0	Sep-13	7,5
26-Apr	5,30	112	103,0	May-17	0,0
18-Jun	14,50	48	125,0	Jun-14	12,5
15-Jun	539,00	45	6,6	Jun-15	0,0
9-Apr	59,00	28	110,0	May-15	15,0
13-Nov	6,50	74	129,0	Nov-12	10,0
18-Jun	12,40	61	129,0	Jul-14	7,5
31-Aug	93,00	84	107,0	Aug-11	0,0
28-Sep	1,40	8	138,0	Nov-16	25,0
2-Feb	6,00	32	134,0	Feb-12	5,0
13-Nov	5,80	49	125,0	Jan-13	0,0
10-Aug	6,30	50	122,0	Aug-11	30,0
8-Mar	1,50	47	113,0	Mar-11	0,0
5-Jan	91,00	89	117,0	Dec-16	25,0
4-Apr	77,80	57	114,0	Apr-17	10,0
20-Jul	2,10	3	156,0	Jul-11	20,0
11-Oct	6,20	5	147,0	Oct-13	5,0
19-Feb	27,00	40	136,0	Feb-16	5,0
12-Feb	73,00	86	114,0	Apr-13	10,0
23-Oct	66,80	94	118,0	Jan-16	20,0
24-Jul	6,40	59	123,0	Aug-17	15,0
4-Jan	30,00	36	151,0	Jan-12	20,0
29-Oct	19,00	43	125,0	Nov-12	0,0
30-Apr	31,00	44	135,0	May-14	2,5
8-Jul	154,00	134	105,0	Jul-13	40,0

7-Aug	5,60	96	114,0	Aug-14	30,0
18-Feb	27,00	67	104,0	Feb-13	20,0
19-Jun	6,10	46	130,0	Jun-15	0,0
27-Mar	15,60	130	43,0	Mar-12	0,0
2-Aug	76,00	97	103,0	Aug-11	0,0
23-Nov	7,30	52	104,0	Oct-15	30,0
16-Jan	9,80	50	119,0	Jan-13	10,0
19-Sep	97,00	75	12,0	Nov-15	10,0
30-Nov	1,90	52	116,0	Dec-16	15,0
17-Aug	23,20	33	141,0	Aug-11	15,0
21-Mar	30,00	86	125,0	Apr-17	5,0
29-Jun	1,30	40	113,0	Oct-12	10,0
28-Sep	21,30	33	120,0	Oct-12	2,5
20-Feb	3,80	83	100,0	Feb-12	15,0
9-Apr	2,10	4	141,0	Apr-14	7,5
30-Nov	42,90	37	139,0		15,0
20-Feb	21,60	24	110,0	Mar-13	60,0
29-Nov	93,00	60	118,0		15,0
8-Jun	21,00	40	136,0	Feb-17	5,0
20-Apr	4,20	43	119,0	Apr-12	10,0
25-Nov	26,60	13	123,0	Jan-15	20,0
8-Aug	223,00	103	115,0	Aug-11	30,0
5-Oct	12,60	39	117,0	Feb-16	15,0
20-May	5,80	28	11,0	Jun-15	15,0
5-Nov	10,30	43	116,0	Nov-14	7,5
20-Sep	12,00	48	128,0	Oct-12	5,0
13-Dec	3,00	38	139,0	Jan-13	10,0
11-Feb	26,00	31	133,0	Apr-15	10012896000,0
16-Oct	0,70	9	127,0	Oct-13	15,0
29-Nov	2,10	25	152,0	Nov-12	7,5
18-Jun	0,70	7	138,0	Apr-12	15,0
6-Oct	25,00	70	108,0	Oct-17	5,0
20-Sep	3,00	9	148,0	Sep-16	2,5
27-Jun	0,70	5	137,0	Jun-13	20,0
13-Jul	0,00	116	126,0	Aug-11	5,0
19-Apr	33,00	33	134,0	May-12	10,0
15-Nov	66,00	83	124,0	Nov-11	0,0
10-Feb	23,00	66	123,0	Apr-16	0,0
19-Jun	5,30	94	126,0	Jun-12	15,0
15-May	14,00	60	129,0	Jun-12	0,0
27-May	19,50	70	133,0	Sep-13	5,0
20-Feb	12,80	38	118,0	Feb-13	20,0
26-Feb	94,00	52	124,0	Apr-16	15,0
1-Jun	40,00	72	117,0	Jul-17	15,0
16-Sep	0,80	27	129,0	Sep-11	0,0
4-May	2,60	28	133,0	Jul-16	5,0
18-Nov	5,90	97	100,0	Nov-13	15,0
4-Aug	12,70	73	105,0	Aug-11	0,0
21-Feb	3,00	43	134,0	Feb-13	10,0
29-Jun	60,00	128	90,0	Jul-17	20,0

8-Jan	0,41	57	135,0	Apr-18	0,0
3-Jan	279,00	73	132,0	Jan-13	0,0
10-Oct	121,00	98	101,0	Oct-13	0,0
8-Jun	15,30	57	117,0	Jun-17	0,0
25-Oct	2,90	6	143,0	Nov-17	15,0

START_DATA_PDN	DOSIS_PDN	OTHER	START_DOSE	FINAL_DIAGNOSIS
Sep-10	50,0	0	50,0	PMR
Jun-11	60,0	0	60,0	LVV
		0	0,0	PMR
Jun-11	15,0	0	15,0	PMR
		0	15,0	PMR
Jun-09	5,0	0	0,0	PMR
Apr-12	30,0	0	30,0	CANCER
Jan-13	30,0	0	30,0	PMR
Apr-12	15,0	0	15,0	CANCER
Nov-11	10,0	0	10,0	PMR
Apr-18	60,0	0	60,0	PMR
		1	30,0	PMR
Mar-12	40,0	0	40,0	PMR
Jun-12	30,0	1	30,0	PMR
May-05		0	0,0	PMR
Jan-15	20,0	0	20,0	PMR
May-11	60,0	0	60,0	LVV
Apr-16	15,0	0	15,0	PMR
Jun-10	30,0	0	30,0	PMR
Jun-12	20,0	0	20,0	PMR
Jun-02	20,0	0	20,0	PMR
Apr-13	15,0	0	15,0	PMR
Jan-11	30,0	0	30,0	PMR
Jun-03	15,0	0	15,0	PMR
	0,0	0	0,0	DEGENERATIVE
Sep-13	20,0	0	20,0	PMR
Jun-15	25,0	0	25,0	PMR/EORA
May-15	15,0	0	15,0	PMR+NEO
May-12	30,0	0	0,0	PMR
Sep-13	15,0	0	15,0	PMR
Aug-11	15,0	0	15,0	PMR
Sep-16	30,0	0	15,0	PMR
Jun-03	2,5	0	0,0	PMR/EORA
	20,0	0	20,0	PMR
May-11	30,0	0	30,0	PMR
May-11	7,5		7,5	PMR
Nov-16	30,0	0	30,0	PMR
Mar-17	10,0	0	10,0	PMR
May-11	30,0	0	30,0	PMR
			30,0	PMR
Mar-14	20,0	0	20,0	PMR
Jun-12		1		LVV
Nov-15	20,0	0	20,0	LVV
Apr-17	15,0	0	15,0	PMR+NEO
Jan-12	20,0	0	20,0	PMR
Nov-12	15,0	0	15,0	PMR
Apr-11	30,0	0	30,0	PMR
Jul-13	40,0	0	40,0	LVV

Apr-14	80,0	0	80,0	PMR
Jan-12	1000,0	0	1000,0	LVV
	0,0	0	0,0	PMR/EORA
Mar-12	15,0	0	15,0	PMR
Aug-11	15,0		15,0	PMR
Oct-15	30,0	0	30,0	LVV
Jun-10	30,0	0	0,0	PMR
Oct-15	10,0	0	10,0	PMR
Jun-11		0		VASCULITIS OEQUEÑO VASO + PMR
Aug-11	15,0	0	15,0	PMR
Jun-10	60,0	0	60,0	PMR
Sep-11	20,0	0	20,0	PMR
Feb-11	30,0	0	30,0	LVV
Jun-10	15,0	0	15,0	PMR
Jun-00				PMR/EORA
Mar-17	15,0	0	15,0	PMR
Oct-09	30,0	0	0,0	PMR
Nov-11	30,0		30,0	PMR
May-16	20,0	0	20,0	PMR
Jun-09	10,0	0	10,0	PMR
Oct-14	20,0	0	20,0	PMR
Aug-11	30,0	0	30,0	PMR/EORA
Mar-15		0		PMR
Mar-15	15,0	0	15,0	PMR
Nov-13	30,0	0	30,0	PMR
Apr-10	30,0	0	30,0	LVV
Jul-12	20,0	0	20,0	PMR
Jan-14	30,0	0	30,0	LVV
Sep-13	15,0	0	15,0	PMR
Jun-10	15,0		15,0	PMR
Jun-10	15,0	0	15,0	PMR
Aug-17		0		PMR/EORA
Jun-15	15,0	0	15,0	PMR
May-13	20,0	0	20,0	PMR
Sep-08	10,0	0	10,0	PMR
Jun-10	15,0	0	15,0	SJOGREN
Nov-11	15,0	0	15,0	PMR
May-16	40,0	0	40,0	LVV
Jun-12	15,0	0	15,0	PMR
Jun-09	20,0	1	20,0	PMR
	5,0		5,0	LVV
Feb-13	20,0	0	20,0	CANCER
Mar-16	15,0	0	15,0	LVV
Nov-16	15,0	0	15,0	PMR
Nov-10	15,0	0	15,0	PMR
Jul-10	20,0	0	20,0	PMR
Sep-13	15,0	0	15,0	LVV
Aug-11	15,0	0	15,0	LVV
Nov-12	20,0	0	20,0	PMR
May-17	20,0	0	20,0	PMR

				PMR
Jan-13	30,0	0	30,0	PMR
Nov-13	1000,0	0	1000,0	LVV
Jul-17		0		PMR
Oct-17	30,0	0	30,0	PMR

AGE	EVOLUTIO	DOSIS_PDN	PMR	SHOULDER_UPTAKE	SUV_H	TBR_H
80,30	0,76	Dosi > 5	Sí	0	1,33	0,892617449664
60,36	40,60	Dosi <= 5	No	0	2,01	1,288461538462
74,17	42,12	Dosi <= 5	Sí	0	3,20	1,828571428571
75,64	5,50	Dosi > 5	Sí	0	3,79	1,559670781893
77,04	0,86	Dosi <= 5	Sí	1	3,45	1,835106382979
68,62	30,26	Dosi <= 5	Sí	0	2,32	1,177664974619
78,41	1,89	Dosi <= 5	No	1	3,06	1,821428571429
79,34	1,69	Dosi > 5	Sí	1	5,01	3,503496503497
87,98	10,50	Dosi <= 5	No	0	2,96	1,429951690821
88,06	6,72	Dosi > 5	Sí	1	3,16	2,038709677419
74,55	-1,69	Dosi <= 5	Sí	1	6,02	2,908212560386
45,63	30,23	Dosi > 5	Sí	0	2,77	1,709876543210
61,82	1,19	Dosi > 5	Sí	0	1,95	1,211180124224
64,90	12,22	Dosi <= 5	Sí	0	2,24	1,600000000000
55,45	212,81	Dosi <= 5	Sí	0	1,75	1,785714285714
71,82	4,07	Dosi > 5	Sí	0	3,79	2,037634408602
83,27	2,88	Dosi <= 5	No	0	2,50	1,633986928105
72,96	7,62	Dosi <= 5	Sí	1	3,47	2,532846715328
65,48	55,89	Dosi <= 5	Sí	1	2,98	1,874213836478
76,23	2,88	Dosi > 5	Sí	1	2,58	2,080645161290
66,17	190,89	Dosi <= 5	Sí	1	2,57	2,072580645161
23,13	2,75	Dosi > 5	Sí	0	2,02	1,216867469880
80,81	9,70	Dosi > 5	Sí	1	4,08	2,205405405405
71,27	124,34	Dosi > 5	Sí	1		
88,60	22,98	Dosi <= 5	No	1		
63,63	12,35	Dosi > 5	Sí	0	2,56	1,620253164557
75,03	0,46	Dosi <= 5	No	1	4,49	3,837606837607
83,25	10,73	Dosi > 5	No	1	4,14	2,688311688312
76,05	41,29	Dosi > 5	Sí	0	2,25	1,184210526316
79,02	10,03	Dosi > 5	Sí	1	2,59	1,962121212121
81,91	2,19	Dosi <= 5	Sí	1	4,04	2,282485875706
71,50	3,61	Dosi > 5	Sí	1	3,21	1,528571428571
59,57	104,67	Dosi <= 5	No	0	1,62	1,065789473684
51,14	103,84	Dosi <= 5	Sí	0	1,87	1,074712643678
72,21	2,88	Dosi > 5	Sí	1	4,13	2,771812080537
14,84	33,44	Dosi <= 5	Sí	0	1,55	1,107142857143
75,93	2,02	Dosi > 5	Sí	0	2,16	1,421052631579
83,00	-0,13	Dosi > 5	Sí	0	1,50	0,666666666667
67,86	2,88	Dosi > 5	Sí	0	2,12	1,367741935484
68,57	4,50	Dosi <= 5	Sí	0	2,40	2,201834862385
72,99	33,61	Dosi <= 5	Sí	1	3,33	2,250000000000
76,26	9,64	Dosi > 5	No	1	3,16	1,628865979381
67,17	8,25	Dosi > 5	No	1	3,30	2,598425196850
81,69	3,58	Dosi > 5	No	0	2,16	0,964285714286
57,76	0,73	Dosi > 5	Sí	1	3,09	1,485576923077
76,46	3,77	Dosi <= 5	Sí	1	7,32	3,641791044776
72,12	35,00	Dosi <= 5	Sí	1	5,05	2,475490196078
60,96	0,53	Dosi > 5	No	1	2,71	1,978102189781

84,11	3,71	Dosi > 5	Sí	0		
76,71	13,34	Dosi > 5	No	0	2,05	1,863636363636
69,41	7,38	Dosi <= 5	No	0	1,97	1,539062500000
79,54	2,95	Dosi <= 5	Sí	1	5,00	3,521126760563
80,91	13,74	Dosi <= 5	Sí	1	4,41	3,801724137931
84,19	1,26	Dosi > 5	No	1	3,51	2,580882352941
59,62	151,99	Dosi > 5	Sí	1	3,40	1,837837837838
67,60	5,17	Dosi > 5	Sí	0	2,05	1,375838926175
78,78	66,09	Dosi > 5	No	1	3,63	2,213414634146
57,60	4,17	Dosi > 5	Sí	1	7,65	3,642857142857
57,43	85,66	Dosi <= 5	Sí	0	2,09	1,060913705584
78,47	15,96	Dosi > 5	Sí	0	2,45	1,282722513089
79,00	20,20	Dosi <= 5	No	0	1,96	1,315436241611
76,02	19,97	Dosi > 5	Sí	0	2,41	1,617449664430
56,51	167,25	Dosi > 5	No	1	3,18	2,134228187919
69,13		Dosi > 5	Sí	0	2,20	1,023255813953
77,82	141,62	Dosi > 5	Sí	0	1,73	1,081250000000
81,80		Dosi > 5	Sí	1		
77,47	8,91	Dosi <= 5	Sí	1	2,58	1,270935960591
70,47	33,84	Dosi > 5	Sí	0	2,03	1,015000000000
66,48	3,64	Dosi > 5	Sí	1	3,00	1,923076923077
64,91	1,19	Dosi > 5	No	1		
72,94	11,49	Dosi > 5	Sí	1	2,84	1,844155844156
80,51	2,81	Dosi > 5	Sí	0	1,57	0,934523809524
69,75	16,82	Dosi > 5	Sí	1	5,36	4,581196581197
72,54	30,40	Dosi <= 5	No	0	2,02	1,262500000000
78,69	17,02	Dosi > 5	Sí	0	2,27	1,086124401914
64,03	17,02	9	No	1	3,60	2,117647058824
82,38	2,22	Dosi > 5	Sí	1	3,63	2,186746987952
72,31	36,39	Dosi > 5	Sí	0	2,90	1,638418079096
73,27	22,55	Dosi > 5	Sí	0	2,77	1,629411764706
80,04	1,72	Dosi <= 5	No	1	5,91	3,110526315789
77,24	9,83	Dosi <= 5	Sí	1	4,07	3,130769230769
67,91	2,42	Dosi > 5	Sí	1	3,69	2,396103896104
85,95	38,74	Dosi <= 5	Sí	1	3,14	2,093333333333
59,58	17,78	Dosi > 5	No	1	3,39	2,778688524590
81,16	6,13	Dosi <= 5	Sí	1	6,80	3,400000000000
81,62	1,36	Dosi <= 5	No	1	2,38	1,515923566879
55,52	5,26	Dosi > 5	Sí	1	3,17	2,830357142857
70,66	33,11	Dosi <= 5	Sí	0	2,77	1,473404255319
76,07	51,75	Dosi <= 5	No	1	3,38	2,139240506329
81,87	16,19	Dosi > 5	No	0	2,16	1,102040816327
73,09	-10,50	Dosi > 5	No	1	2,89	1,596685082873
78,71	-16,09	Dosi > 5	Sí	1	5,09	4,072000000000
76,38	11,13	Dosi <= 5	Sí	1	3,75	1,903553299492
74,59	73,25	Dosi <= 5	Sí	0	1,57	1,154411764706
72,74	5,26	Dosi > 5	No	0	2,04	1,186046511628
81,01	6,09	Dosi <= 5	No	1	3,58	2,983333333333
79,15	7,45	Dosi > 5	Sí	0	1,44	1,469387755102
76,55	3,94	Dosi > 5	Sí	1	7,27	3,350230414747

56,06	12,35	Dosi <= 5	Sí	1	3,38	1,769633507853
67,70	1,72	Dosi <= 5	Sí	1	4,60	3,333333333333
65,52	1,49	Dosi <= 5	No	0	2,27	1,247252747253
52,85	2,48	Dosi <= 5	Sí	1	3,36	2,452554744526
65,25	4,50	Dosi > 5	Sí	1	5,75	3,965517241379

WRIST_UPTAKE	SUV_C	TBR_C	SUV_A	TBR_A	VASCULITIS
1	3,45	2,315436241611			0
0	1,87	1,198717948718			0
0	2,10	1,200000000000			0
0	2,20	0,905349794239			0
0	2,40	1,276595744681			0
0	2,31	1,172588832487			0
1	4,19	2,494047619048			0
1	4,62	3,230769230769	4,58	3,202797202797	0
0	2,17	1,048309178744			0
0	1,68	1,083870967742			0
1	5,80	2,801932367150	3,95	1,908212560386	0
0	3,00	1,851851851852			0
0	2,87	1,782608695652			0
0	1,68	1,200000000000			0
0	1,75	1,785714285714			0
0	3,40	1,827956989247			0
1	3,78	2,470588235294			1
1	3,64	2,656934306569	3,70	2,700729927007	0
0	1,90	1,194968553459			0
1	2,55	2,056451612903			0
1	2,62	2,112903225806			0
0	2,59	1,560240963855			0
1	3,30	1,783783783784			0
0					0
1					0
0	2,69	1,702531645570			0
0	3,21	2,743589743590	5,38	4,598290598291	0
0	2,39	1,551948051948			0
0	1,95	1,026315789474			0
1	3,46	2,621212121212			0
1	5,47	3,090395480226	3,44	1,943502824859	0
1	2,90	1,380952380952			0
0	1,67	1,098684210526			0
0	1,81	1,040229885057			0
0	2,42	1,624161073826			0
0	1,48	1,057142857143			0
0	2,13	1,401315789474			0
0	2,10	0,933333333333			0
0	2,27	1,464516129032			0
0	1,26	1,155963302752			0
0	1,66	1,121621621622			0
0	2,45	1,262886597938			0
0	2,47	1,944881889764	3,13	2,464566929134	1
0	1,76	0,785714285714			0
1	2,30	1,105769230769			0
1	5,16	2,567164179104	4,80		0
0	2,68	1,313725490196			0
0	1,75	1,277372262774	5,25	3,832116788321	0

0					0
0	1,56	1,418181818182			0
0	1,86	1,453125000000			0
1	8,01	5,640845070423	4,26	3,000000000000	0
0	2,27	1,956896551724	3,94	3,396551724138	0
1	3,14	2,308823529412	2,51	1,845588235294	1?
0	2,59	1,400000000000			0
0	1,62	1,087248322148			0
0	1,80	1,097560975610			0
1	7,69	3,661904761905	2,83	1,347619047619	0
0	3,21	1,629441624365			0
0	2,64	1,382198952880			0
0	1,48	0,993288590604			1
0	2,24	1,503355704698			0
1	4,54	3,046979865772			0
0	3,16	1,469767441860	4,70	2,186046511628	1
0	1,40	0,875000000000			0
1					0
1	3,86	1,901477832512			0
0	2,13	1,065000000000			0
1	3,72	2,384615384615			0
1					0
0	2,03	1,318181818182			0
0	1,52	0,904761904762			0
1	5,40	4,615384615385	6,87	5,871794871795	0
0	2,41	1,506250000000			1
0	2,30	1,100478468900			0
1	3,56	2,094117647059	4,10	2,411764705882	0
1	3,25	1,957831325301			0
0	2,09	1,180790960452			0
0	1,53	0,900000000000			0
1	4,33	2,278947368421	3,15	1,657894736842	1
1	3,32	2,553846153846	4,54	3,492307692308	0
1	3,05	1,980519480519	2,75	1,785714285714	0
0	1,42	0,946666666667			0
0	1,94	1,590163934426	2,17		0
1	3,76	1,880000000000			0
1	7,60	4,840764331210	3,20	2,038216560510	1
0	2,46	2,196428571429			0
0	2,05	1,090425531915			0
1	3,37	2,132911392405			1
0	2,20	1,122448979592	3,11	1,586734693878	1
0	2,24	1,237569060773			0
1	5,05	4,040000000000	3,89	3,112000000000	0
0	2,33	1,182741116751			0
0	1,60	1,176470588235			0
0	2,22	1,290697674419			0
1	4,70	3,916666666667			0
0	1,06	1,081632653061			0
0	3,11	1,433179723502			0

1	3,19	1,670157068063			0
1	3,63	2,630434782609	4,08	2,956521739130	0
0	2,57	1,412087912088			1
1	2,97	2,167883211679	2,51	1,832116788321	0
1	3,90	2,689655172414	3,49	2,406896551724	0

AORTICA_UPTAKE	TBR_Aortic	Act_max_cava	Act_med_cava	SPINE_C
1,86	1,248322147651	1,72	1,49	1
2,61	1,673076923077	1,83	1,56	0
2,90	1,657142857143	2,30	1,75	0
3,36	1,382716049383	2,66	2,43	0
2,69	1,430851063830	2,28	1,88	1
2,45	1,243654822335	2,77	1,97	0
2,40	1,428571428571	2,00	1,68	0
1,97	1,377622377622	1,76	1,43	1
2,28	1,101449275362	2,44	2,07	0
2,69	1,735483870968	1,86	1,55	0
2,54	1,227053140097	2,39	2,07	1
2,50	1,543209876543	1,83	1,62	0
2,40	1,490683229814	1,95	1,61	0
2,40	1,714285714286	1,75	1,40	0
1,87	1,908163265306	1,21	0,98	0
3,55	1,908602150538	2,42	1,86	0
6,16	4,026143790850	1,77	1,53	0
2,11	1,540145985401	1,69	1,37	1
2,44	1,534591194969	1,90	1,59	0
1,73	1,395161290323	1,55	1,24	0
1,96	1,580645161290	1,52	1,24	1
2,36	1,421686746988	1,94	1,66	1
3,12	1,686486486486	2,47	1,85	0
				0
				0
2,23	1,411392405063	1,99	1,58	0
3,16	2,700854700855	2,02	1,17	1
2,29	1,487012987013	1,74	1,54	0
2,59	1,363157894737	2,30	1,90	0
2,79	2,113636363636	1,71	1,32	0
2,48	1,401129943503	2,20	1,77	0
2,89	1,376190476190	2,51	2,10	1
2,46	1,618421052632	1,98	1,52	0
2,40	1,379310344828	1,99	1,74	0
2,43	1,630872483221	1,88	1,49	0
1,96	1,400000000000	1,62	1,40	0
2,17	1,427631578947	1,99	1,52	0
2,60	1,155555555556	2,55	2,25	0
2,35	1,516129032258	1,87	1,55	0
1,97	1,807339449541	1,31	1,09	0
3,07	2,074324324324	1,70	1,48	0
2,84	1,463917525773	2,27	1,94	1
2,73	2,149606299213	1,64	1,27	1
3,62	1,616071428571	2,63	2,24	0
2,73	1,312500000000	2,38	2,08	0
3,29	1,636815920398	2,52	2,01	1
2,80	1,372549019608	2,56	2,04	0
2,37	1,729927007299	1,53	1,37	1

				0
2,34	2,127272727273	1,37	1,10	0
2,08	1,625000000000	1,50	1,28	0
1,92	1,352112676056	1,70	1,42	1
1,83	1,577586206897	1,36	1,16	1
4,23		1,78	1,36	1
2,42	1,308108108108	2,21	1,85	0
2,25	1,510067114094	1,83	1,49	1
2,55	1,554878048780	2,23	1,64	0
3,13	1,490476190476	2,48	2,10	0
3,00	1,522842639594	2,27	1,97	0
2,65	1,387434554974	2,18	1,91	1
2,62	1,758389261745	1,93	1,49	0
1,97	1,322147651007	1,82	1,49	0
2,48	1,664429530201	1,99	1,49	0
3,75	1,744186046512	2,45	2,15	1
2,24	1,400000000000	1,88	1,60	0
				1
2,62	1,290640394089	2,31	2,03	1
2,83	1,415000000000	2,46	2,00	0
2,18	1,397435897436	1,83	1,56	0
				0
2,02	1,311688311688	1,93	1,54	0
2,54	1,511904761905	2,25	1,68	0
2,62	2,239316239316	1,86	1,17	1
3,53	2,206250000000	1,82	1,60	0
2,60	1,244019138756	2,48	2,09	0
2,70	1,588235294118	2,09	1,70	1
3,04	1,831325301205	2,17	1,66	0
2,60	1,468926553672	2,07	1,77	0
2,56	1,505882352941	2,15	1,70	0
4,24	2,231578947368	2,29	1,90	1
2,11	1,623076923077	1,51	1,30	1
2,34	1,519480519481	1,79	1,54	0
2,56	1,706666666667	2,03	1,50	0
2,51	2,057377049180	1,67	1,22	0
2,67	1,335000000000	2,39	2,00	1
3,79	2,414012738854	1,86	1,57	1
1,86	1,660714285714	1,70	1,12	0
2,45	1,303191489362	2,21	1,88	0
3,22	2,037974683544	1,82	1,58	0
3,32	1,693877551020	2,15	1,96	1
2,45	1,353591160221	2,25	1,81	1
1,84	1,472000000000	1,41	1,25	1
3,35	1,700507614213	2,22	1,97	1
1,83	1,345588235294	1,67	1,36	1
2,65	1,540697674419	2,00	1,72	0
2,34	1,950000000000	1,39	1,20	1
1,81	1,846938775510	1,15	0,98	0
3,14	1,447004608295	2,75	2,17	0

3,16	1,654450261780	2,44	1,91	0
2,32	1,681159420290	1,89	1,38	0
3,18	1,747252747253	2,16	1,82	1
2,43	1,773722627737	1,89	1,37	0
2,30	1,586206896552	1,64	1,45	0

SPINE_D	SPINE_L	ISQUIAT	SUV_bursae	TBR_bursae
1	1	0	4,52	3,03355704698
0	0	0		
0	1	0	5,50	3,14285714286
0	0	0		
0	1	1	3,94	2,09574468085
0	1	0	3,21	1,62944162437
0	0	0		
0	1	1	1,99	1,39160839161
0	0	0		
0	0	0		
0	1	0	5,34	2,57971014493
0	0	0		
0	0	0		
0	0	0		
0	0	0		
0	0	0		
0	1	0	4,45	2,90849673203
0	1	1	5,07	3,70072992701
0	0	0		
0	1	1	2,64	2,12903225806
0	1	1	2,57	2,07258064516
0	1	1	2,82	1,69879518072
0	0	0		
0	0	0		
0	0	0		
0	0	0		
0	0	1	4,32	3,69230769231
0	0	0		
0	0	0		
0	0	1	3,45	2,61363636364
0	0	0		
0	0	1	2,47	1,17619047619
0	0	0		
0	0	0		
0	0	0		
0	0	0		
0	0	0		
0	0	0		
0	0	0		
0	0	0		
0	0	0		
0	1	0	3,32	1,71134020619
0	1	1	2,98	2,34645669291
0	0	0		
0	1	1	2,89	1,38942307692
0	1	1	4,13	2,05472636816
0	0	0		
0	1	0	3,46	2,52554744526

0	0	0		
0	0	0		
0	0	0		
0	1	1	2,96	2,08450704225
1	1	0	4,41	3,80172413793
0	1	0	5,54	4,07352941176
0	0	0		
0	0	0	2,63	1,76510067114
0	0	0		
0	0	0		
0	0	0		
0	0	0		
0	0	0		
0	0	0		
0	1	1	4,58	3,07382550336
0	1	1	4,86	2,26046511628
0	0	0		
0	1	1		
0	1	1	4,20	2,06896551724
0	0	0		
0	0	1	3,98	2,55128205128
0	0	1		
0	0	0		
0	0	0		
0	1	1	5,29	4,52136752137
0	0	0		
0	0	0		
0	1	1	4,22	2,48235294118
0	1	1	3,33	2,00602409639
0	0	0		
0	0	0		
0	1	1	4,73	2,48947368421
0	0	1	3,59	2,76153846154
0	0	1	3,40	2,20779220779
0	0	0		
0	0	0		
0	1	1	4,00	2,00000000000
0	1	1	8,49	5,40764331210
0	0	0		
0	0	0		
0	1	1	3,47	2,19620253165
1	1	1	2,61	1,33163265306
0	1	0	3,86	2,13259668508
1	1	1	6,69	5,35200000000
0	1	1	3,26	1,65482233503
0	0	1	2,44	1,79411764706
0	0	0		
0	1	1	3,39	2,82500000000
0	0	0		
0	0	1	5,93	2,73271889401

0	1	0	3,20	1,67539267016
0	1	1	3,33	2,41304347826
0	1	1	3,51	1,92857142857
0	1	1	4,37	3,18978102190
0	0	0		
